# Effect of *in vitro* storage duration on measured mechanical properties of brain tissue

**DOI:** 10.1038/s41598-018-19687-2

**Published:** 2018-01-19

**Authors:** Wei Zhang, Li-fu Liu, Yue-jiao Xiong, Yi-fan Liu, Sheng-bo Yu, Cheng-wei Wu, Weihong Guo

**Affiliations:** 1 State Key Laboratory of Structural Analysis for Industrial Equipment, Department of Engineering Mechanics, Dalian University of Technology, Dalian, 116024 China; 2 Department of Anatomy, College of Basic Medical Sciences, Dalian Medical University, Dalian, 116044 China; 3 Department of Industrial and Systems Engineering, Rutgers-The State University of New Jersey, Piscataway, NJ 08854 USA

## Abstract

Accurate characterization of the mechanical properties of brain tissue is essential for understanding the mechanisms of traumatic brain injuries and developing protective gears or facilities. However, how storage conditions might affect the mechanical properties of brain tissue remains unclear. The objective of this study is to investigate the effect of *in vitro* storage duration on the mechanical performance of brain tissue since measurements are usually carried out *in vitro*. Differential Scanning Calorimetry (DSC) measurements and uniaxial compression mechanical experiments are carried out. The results indicate that, for brain tissue stored at 1 °C without any liquid medium, the bio-molecular interactions and the mechanical strength of both white and grey matter deteriorate with prolonged storage duration. Transmission Electron Microscopy (TEM) results reveal the degeneration of myelin sheaths and the vacuolization of cristae with prolonged storage duration, suggesting that the *in vitro* storage duration should be carefully controlled. The findings from this study might facilitate the development of guidelines and standards for the *in vitro* storage of brain tissue.

## Introduction

Traumatic brain injury (TBI) is sudden damage to the brain caused by a blow or jolt to the head^1^. Common causes include car or motorcycle crashes, falls, sports injuries, and assaults. TBI is the leading cause of death and disability in young people^2^. According to the Centers for Disease Control and Prevention (CDC), about 2.5 million emergency room visits, 282,000 hospitalizations, and 50,000 deaths occur each year as a result of a TBI in the United States^3^. To gain a better understanding of the mechanisms of TBI and develop protective devices, there has been increasing interest in modeling the mechanical response of brain tissue. There is no doubt that the accurate mechanical property data of brain tissue is of critical importance to validate the constitutive models and therefore to ensure the reliability of simulation results.

There has been a lot of research studying the *in vitro* mechanical properties of brain tissue^4–10^. Although there is a consensus among researchers that the brain tissue demonstrates typical viscoelastic behavior, data reported in literature show large discrepancies even in the linear viscoelastic regime. There are no universally acceptable data available for the mechanical properties of brain tissue, due to the inhomogeneous nature of brain tissue (e.g., anatomic region, anisotropy, and species and age of the animal) and the broad range of testing conditions and protocols (e.g., temperature, humidity, pre-condition, and manner of applying loading)^11–13^.

The effect of storage conditions after dissection – including storage duration and temperature – on the mechanical response of brain tissue is an important issue but remains unclear. On the effect of storage duration, Metz *et al*.^14^ observed a decrease in the tissue response to inflation of the balloon catheter as the tissue is tested from live to 3/4 h post-mortem; Nicolle *et al*.^13^ observed an increase in the shear modulus when the post-mortem time was increased from 24 to 48 h. To the contrary, McElhaney *et al*.^15^ reported that there was no obvious change in the mechanical response of the tissue within 15 h post-mortem. On the effect of storage temperature, Zhang *et al*.^16^ found that the brain tissue stored at 37 °C is stiffer than that stored at the ice-cold temperature. Rashid *et al*.^17^ compared the brain tissue stored at ice-cold temperature, room temperature (22 °C), and body temperature (37 °C), and reported that the initial elastic modulus decreases as the temperature increases, and that the difference can be over 2 folds. These studies, however, were carried out in different testing conditions and on inhomogeneous brain tissue samples. The findings from these studies are not able to provide a standard for brain tissue storage conditions. As a result, various levels of storage temperature and duration have been used in brain tissue studies. The most widely adopted procedure is storing the brain tissue at 0-5 °C for a few hours or even up to four days^11–27^. However, little is known about whether or not the storage duration at this temperature range will affect the mechanical response of brain tissue^28,29^.

In this study, the effect of storage duration on the mechanical response of sheep brain tissue is examined. Brain tissue samples (both white and grey matter samples) were stored at 1.0 ± 1.0 °C in *vitro* for various durations and their mechanical performance was studied. Differential Scanning Calorimetry (DSC) measurements of the samples were analyzed to characterize the thermal behavior of brain tissue. With the attempt to understand the effect of prolonged storage duration on the mechanical properties, the brain tissue slices were also analyzed using Transmission Electron Microscope (TEM).

## Results and Discussion

### Characterization of thermal behavior with DSC

**Figure 1:**
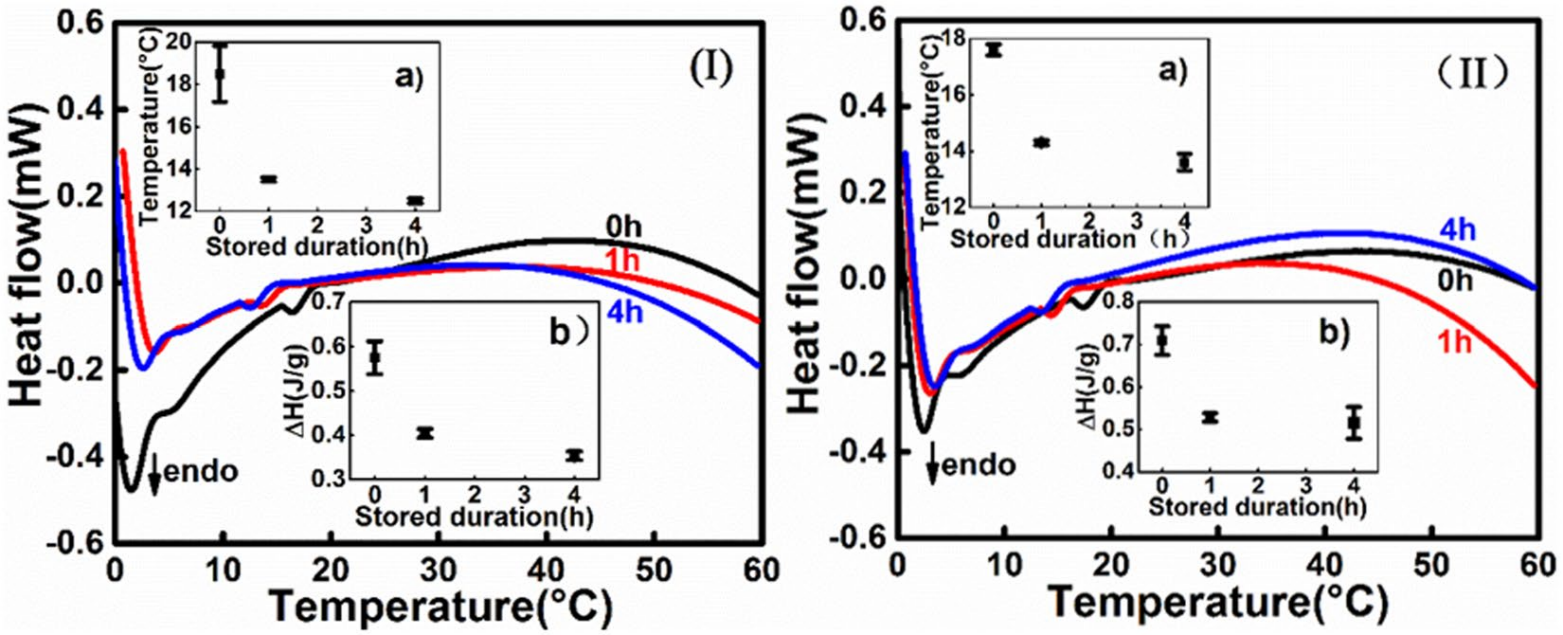
DSC curves on heat flow, temperature, and duration. (**I**) DSC curves of white matter. (**II**) DSC curves of grey matter. Inset (a) shows the corresponding peak temperature at each duration. Inset (b) shows the enthalpy at each duration. Error bars in insets represent the standard deviation from 4 individual samples.

Differential Scanning Calorimetry (DSC) thermogram provides a unique and sensitive signature for physicochemical transformations in bio-molecules and has been proved to be an efficient approach for demonstrating the structural changes in biological tissues^30,31^. To characterize the effect of storage duration on the thermal behavior of brain tissue, an aluminum pan with sealed samples of white and grey matter was cooled to 1 °C and maintained there for 0 h, 1 h and 4 h. Then the temperature was increased linearly to 60 °C. The difference in the amount of heat required to increase the temperature of the brain tissue and the reference is measured as a function of temperature. The recorded DSC curves are given in Fig. 1. The black, red, and blue curves correspond to the heat flow responses for 0 h, 1 h, and 4 h storage, respectively. Each curve shows a representative result among the four individual samples in each setting.

Figure 1(I) demonstrates that white matter samples stored at 1 °C for 0 h, 1 h and 4 h exhibit a distinguishable endothermal domain in 10-20 °C. The peak temperature and the corresponding calorimetric enthalpy change (*∆H*, normalized for net mass) are plotted in insets (a) and (b), respectively. Figure 1(I-a) shows that for white matter stored for 0 h, the thermal transition process takes place at higher temperatures (18.5 ± 1.3 °C, mean ± s.d.) than stored for 1 h or 4 h (13.6 ± 0.1 °C for 1 hr and 12.5 ± 0.1 °C for 4 hr storage, mean ± s.d.). To determine whether there are any statistically significant differences between the peak temperatures of these three groups, the one-way analysis of variance (ANOVA) was carried out. The level of significance for all the significance analyses hereafter is defined as 0.05. A *p* value of 0.0201 was obtained for the peak temperature data in Fig. 1(I-a), suggesting the peak temperature responses from the 0 h, 1 h, and 4 h storage settings are not equal. From Fig. 1(I-b), we observe that the enthalpy also decreases with the prolonged storage duration, 0.58 ± 0.04 J/g for 0 h, 0.41 ± 0.01 J/g for 1 h and 0.35 ± 0.01 J/g (mean ± s.d.) for 4 h storage. The *p* value from one-way ANOVA is 0.0242 for the enthalpy data in Fig. 1(I-b), indicating the mean enthalpy responses from the 0 h, 1 h, and 4 h storage settings are not equal.

Similar observations can be made from Fig. 1(II) for grey matter. After storing the samples at 1 °C for 0 h, 1 h and 4 h, the thermal event occurs at 17.6 ± 0.2 °C, 14.3 ± 0.1 °C and 13.6 ± 0.3 °C (mean ± s.d.), respectively, and the enthalpy reduces from 0.71 ± 0.03 J/g to 0.53 ± 0.01 J/g and then to 0.51 ± 0.04 J/g (mean ± s.d.). For the peak temperature data in Fig. 1(II-a), one-way ANOVA gives a *p* value smaller than 0.0001; for the enthalpy data in Fig. 1(II-b), one-way ANOVA gives a *p* value of 0.0304. These results indicate that the differences are significant.

To further understand the differences in peak temperatures (or enthalpies) caused by different storage durations, we performed pairwise comparisons to compare the peak temperatures (or enthalpies) after storage of 0 h, 1 h, and 4 h to one another. The comparison results are presented in Tables 1 and 2. It is noted that the *p* values when comparing the peak temperatures (or enthalpies) between the 0 h storage duration group and the 1 h storage duration group are all less than 0.05, indicating statistically significant differences in the samples’ thermal behaviors when stored at 0 h versus 1 h. We also notice statistically significant differences in the samples’ thermal behaviors (peak temperatures and enthalpies) when stored at 0 h versus 4 h (pairwise comparison *p* values less than 0.05). The pairwise comparison *p* value between the 1 h storage duration group and the 4 h storage duration group is greater than 0.05, indicating there is not statistically significant differences in the two groups. The same results are observed for both white and grey matter samples.

**Table 1:** Pairwise comparisons of peak temperature and enthalpy of white matter.

Storage duration	Storage duration	*p*-value	
		peak temperature	enthalpy
0	1	0.016	0.005
0	4	0.005	0.001
1	4	0.334	0.079

**Table 2:** Pairwise comparisons of peak temperature and enthalpy of grey matter.

Storage duration	Storage duration	*p*-value	
		peak temperature	enthalpy
0	1	0.049	<0.0001
0	4	0.012	<0.0001
1	4	0.316	0.079

Results shown in Fig. 1 lead to the conclusion that, for both white and grey matter, the characteristic peak temperature drops with the prolongation of storage duration and the entropy also decreases with prolonged storage duration. Previous studies from Skrzyński^32^ and Ignatieva^33^ have concluded that the transition temperature can be related to the cross-linking density of bio-molecules. The higher the transition temperature, the higher cross-linking density and the higher mechanical strength. Ferencz *et al*.^34^ commented that the decrease in calorimetric enthalpy is a definite sign of structural damage in biological tissues.

Hence, the DSC results on brain tissue thermal behavior indicate that the prolonged storage at 1 °C may cause the mechanical strength of brain tissue to deteriorate.

### Mechanical performance in compression

**Figure 2:**
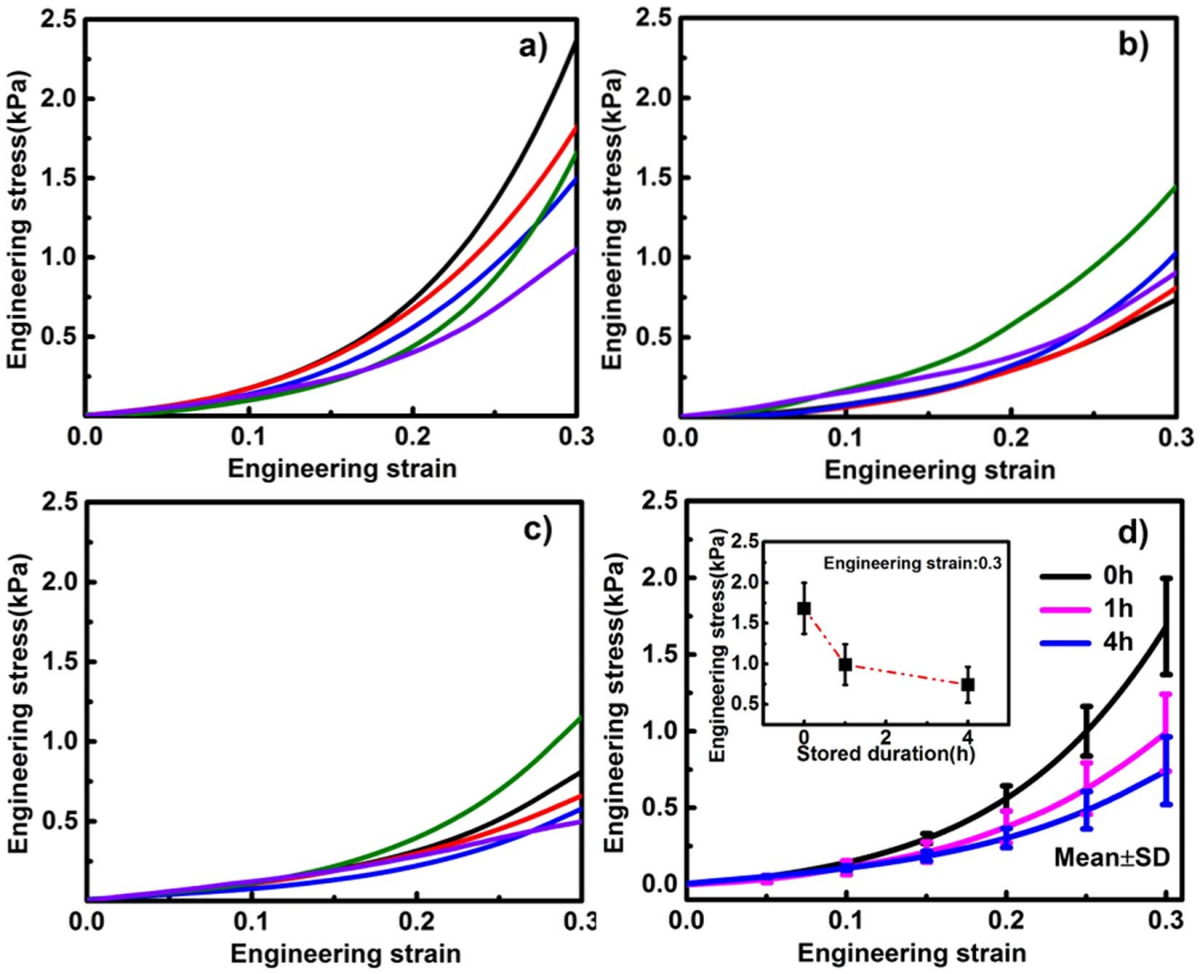
Engineering stress-strain curves of white matter in compression after storage of 0 h, 1 h and 4 h. (**a**) Five individual samples after storage of 0 h. (**b**) Five individual samples after storage of 1 h. (**c**) Five individual samples after storage 4 h. (**d**) The mean values and standard deviations (s.d.) from 5 individual samples, mean ± s.d., for the samples after storage of 0 h, 1 h and 4 h. Inset shows the mean values with ± s.d. for the 5 individual samples at the strain of 0.3.

**Figure 3:**
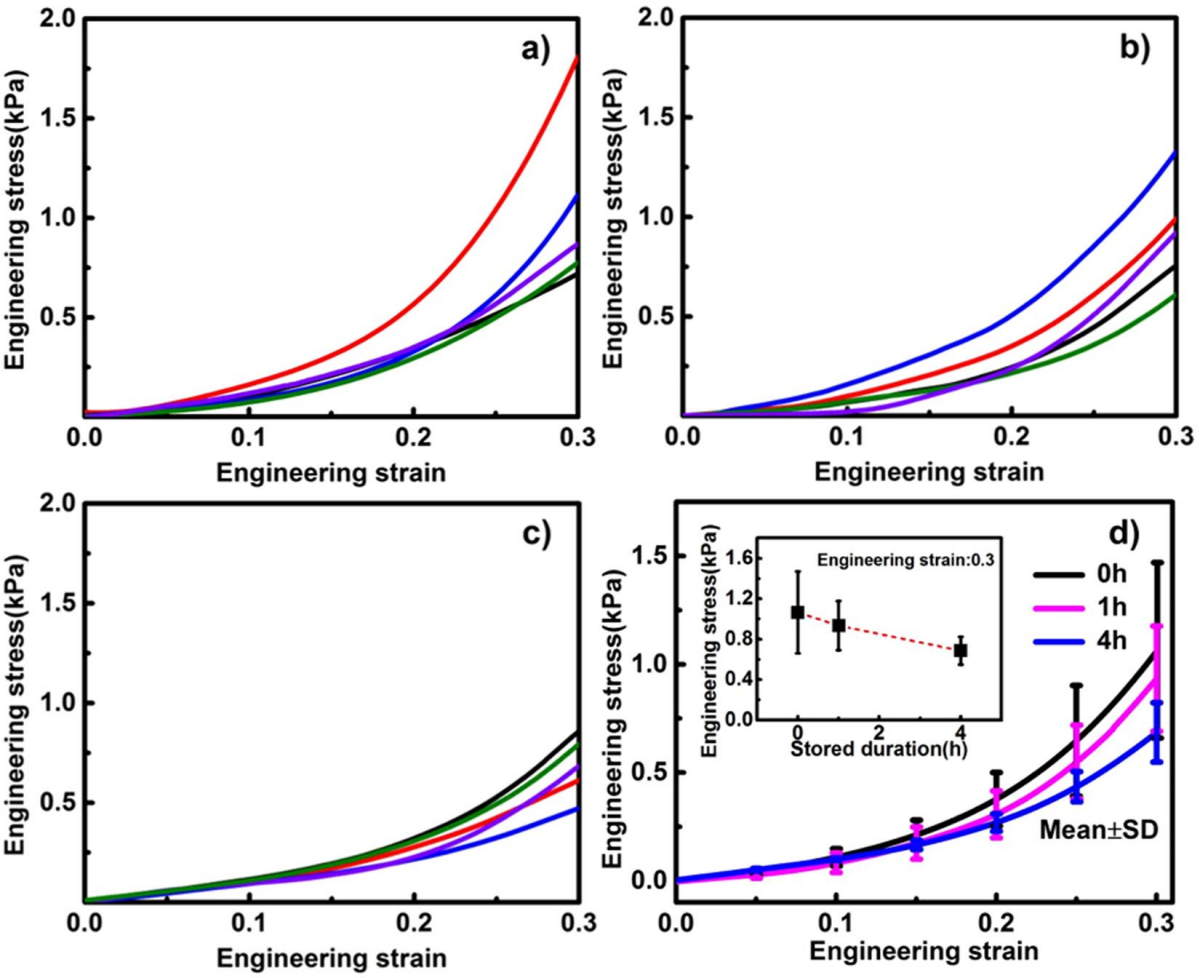
Engineering stress-strain curves of grey matter in compression after storage of 0 h, 1 h and 4 h. (**a**) Five individual samples after storage of 0 h. (**b**) Five individual samples after storage of 1 h. (**c**) Five individual samples after storage 4 h. (**d**) The mean values and standard deviations (s.d.) from 5 individual samples, mean ± s.d., for the samples after storage of 0 h, 1 h and 4 h. Inset shows the mean values with ± s.d. for the 5 individual samples at the strain of 0.3.

The DSC analyses above indicate the deterioration of brain tissue stiffness due to the prolongation of storage duration. But this finding is deduced from evidence at the molecular level. To characterize the effect of low-temperature storage duration on the stiffness of brain tissue at the macro level, the samples of white and grey matter were compressed at a strain rate of 0.01/s after stored at 1 °C for 0 h, 1 h and 4 h. The engineering stress-strain curves are given in Figs 2 and 3.

Figures 2 and 3 indicate that the relationship between engineering stress and strain is non-linear, for both white and grey matter. For the white matter at the strain of 0.3 (Fig. 2d and the inset), the stress drops from 1.68 ± 0.31 kPa after 0 h storage to 0.99 ± 0.25 kPa after 1 h storage and then to 0.74 ± 0.22 kPa (mean ± s.d.) after 4 h storage, indicating a significant decrease in the stiffness of brain tissue due to the prolongation of storage duration. For the data in the inset of Fig. 2d, we obtained a *p* value of 0.0029, confirming that the decrease in stiffness is statistically significant at the strain of 0.3. We consider the storage duration and engineering strain as two factors in the experiment. Hence, we formulated the two-way ANOVA to test whether different levels of the storage duration or engineering strain make a difference in the stress response. The storage duration has 3 levels, 0 h, 1 h, and 4 h; the strain has 31 levels, ranging from 0.00 to 0.30 in increments of 0.01. The *p* value for the duration factor is less than 0.0001. The *p* value shows that there is a significant decrease in the engineering stress of white matter samples due to prolonged storage duration.

A similar decreasing trend can be observed for the grey matter. At the strain of 0.3 (Fig. 3d and the inset), the engineering stresses of grey matter after storage of 0 h, 1 h and 4 h are 1.06 ± 0.41 kPa, 0.93 ± 0.24 kPa and 0.69 ± 0.14 kPa (mean ± s.d.), respectively, indicating a decreasing trend in the engineering stress of grey matter due to the prolongation of storage duration. With 3 levels of storage duration (0 h, 1 h, and 4 h) and 31 levels of strain (0.00 to 0.30 in increment of 0.01), we formulated the two-way ANOVA to test whether different levels of the storage duration or engineering strain make a difference in the stress response. The *p* value for the duration factor is less than 0.0001. The *p* value shows that there is a significant decrease in the engineering stress of grey matter samples due to prolonged storage duration.

To further understand the differences in engineering stresses caused by different storage durations, we performed pairwise comparisons to compare the engineering stresses at the strain of 0.3 after storage of 0 h, 1 h, and 4 h to one another. The comparison results are presented in Tables 3 and 4. For the white matter, presented in Table 3, there are statistically significant differences between the engineering stresses from the 0 h storage duration group and the 1 h storage duration group (*p* value < 0.0001); there are also significant differences between the engineering stresses from the 0 h storage duration group and the 4 h storage duration group (*p* value < 0.0001). The pairwise comparison *p* value between the 1 h storage duration group and the 4 h storage duration group is greater than 0.05, indicating there is not statistically significant differences in the two groups. For the grey matter, all the pairwise comparisons give *p* values less than 0.05 which are presented in Table 4 indicating statistically significant differences between any two groups.

**Table 3:** Pairwise comparisons of stiffness of white matter.

Storage duration	Storage duration	*p*-value
0	1	<0.0001
0	4	<0.0001
1	4	0.115

**Table 4:** Pairwise comparisons of stiffness of grey matter.

Storage duration	Storage duration	*p*-value
0	1	0.001
0	4	<0.0001
1	4	0.009

Furthermore, Figs 2 and 3 indicate that white matter is stiffer than grey matter after stored for the same duration. This is consistent with observations from Budday *et al*.^35^ and Jin *et al*.^36^. The difference in the stiffness between white matter and grey matter is caused by the inherent difference in the components of the white and grey matter: Grey matter consists of a mixture of neuronal cell bodies, their unmyelinated processes and neuroglia, whereas white matter is composed of myelinated axonal fibers surrounded by supporting cells (oligodendrocytes, astrocytes, ependyma and microglia) and blood vessels^37^. Such a fibrous structure confers to the stronger mechanical strength of white matter.

### Characterization of formation of ice with DSC

**Figure 4:**
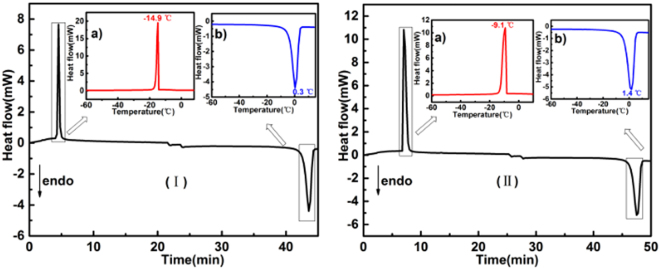
DSC curves of the formation and melting of ice in white matter (**I**) and grey matter (**II**) against time. Note that the temperature is decreased or increased linearly with time. Insets (a) and (b) show the DSC curves of the formation and melting of ice against temperature.

It is estimated that the brain contains about 79% water by weight^38^. This easily makes one to infer that storing brain tissues at low temperatures may cause ice formation. The formation of ice may also exert damaging effects to biological materials through the dehydration and concentration of solutes^39^. This in turn brings about irreversible conformational changes in brain tissues, such as dissociation of macro-molecular complexes of organellar membranes, formation of disulfide bonds in protein, reduction of DNA and protein synthesis. To investigate this issue, the white and grey matter samples were sealed in an aluminum pan, cooled to −100 °C, and then heated to 10 °C. The DSC thermograms are shown in Fig. 4.

For white matter (Fig. 4I), two significant thermal processes are observed in the temperature range investigated: an exothermic peak at −14.9 °C and an endothermic peak at 0.3 °C. The former may correspond to the formation of ice and the latter may result from the melting of ice. With respect to grey matter (Fig. 4II), the exothermic and endothermic peaks are observed at −9.1 °C and 1.4 °C, respectively. These results indicate that although water in both white and grey matter may form ice as the temperature drops, this phase transition from water to ice occurs at a much lower temperature, −14.9 °C for the white matter and −9.1 °C for the grey matter. For brain tissues stored at 1 °C, it is very unlikely to form a large amount of ice; therefore, the damaging effect of ice formation on brain tissue is expected to be insignificant.

### TEM observation

**Figure 5:**
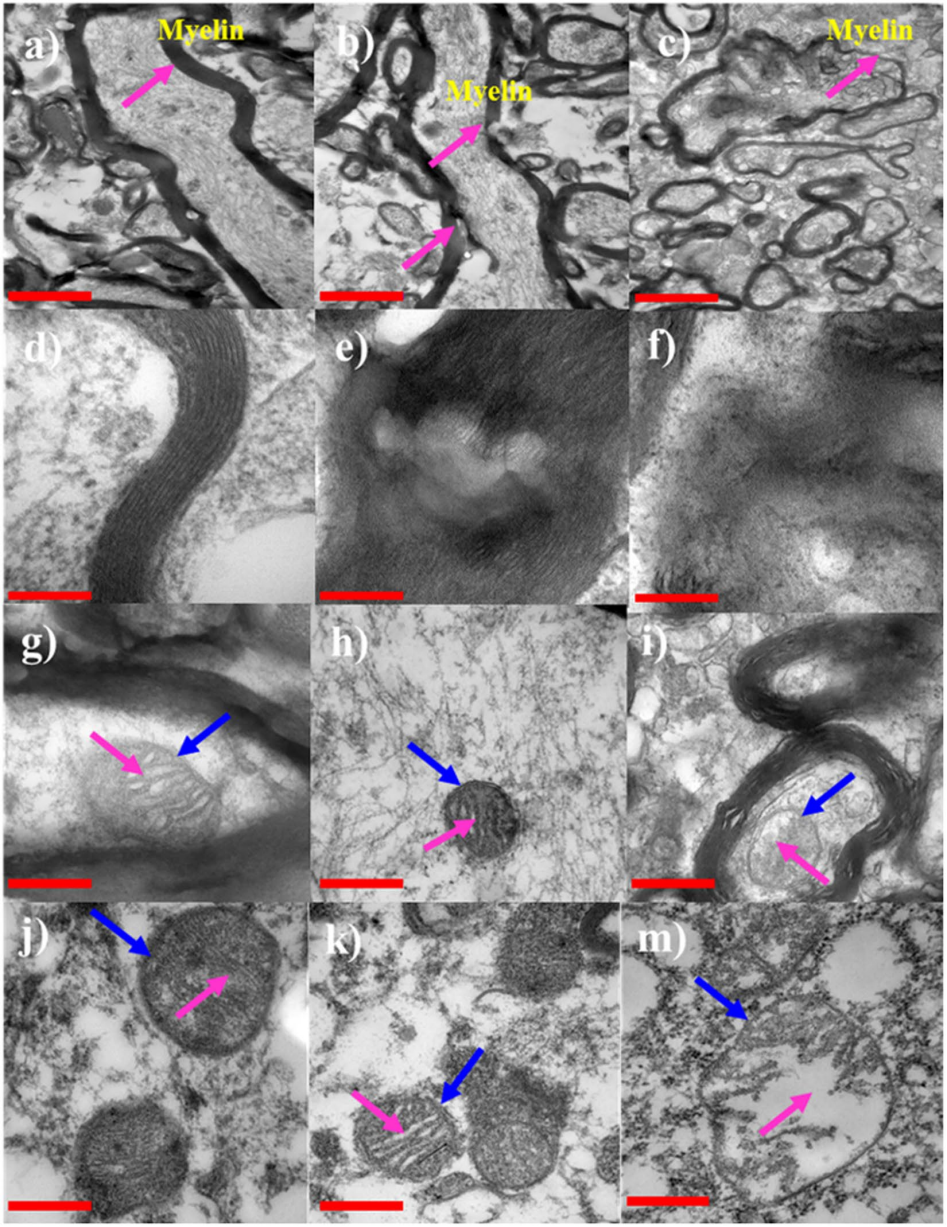
TEM micrograph of the cross-section of the myelinated axon from white matter, stored for 0 h (**a**), 1 h (**b**), and 4 h (**c**), scale bar 1 μm, and the corresponding high magnification images of the myelin sheaths (**d**,**e**,**f**, respectively), scale bar 200 nm. Note that the myelin sheath has a lamellated structure. TEM micrograph of mitochondria in axon (**g**,**h**,**i**) and mitochondria in astrocyte (**j**,**k**,**m**), scale bar 400 nm. Note the variation of cristae.

White matter parts of the brain are characterized by a high density of myelinated axons passing through them. The myelin sheaths of axons give the white matter its light color. Myelin is a fatty substance that wraps around axons and serves to increase the speed of electrical communication between neurons^40,41^. Recently, it has been recognized that the white matter myelin content also affects the mechanical properties of biological tissue. To understand the effect of storage duration on cell organelles, the cross-sections of myelinated axons were observed using TEM (Transmission Electron Microscopy) and the images are shown in Fig. 5. As Fig. 5a illustrates, when storing the brain tissue sample for 0 h, the axon is surrounded by multilamellar structure of myelin and the gaps between the myelin sheathes along axons appear at evenly spaced intervals, approximately 11 nm (Fig. 5d). After stored for 1 h (Fig. 5b), the sheath maintains its tight multilamellar structure, albeit with certain disruption. Localized folding and attenuation can be observed, as indicated by the magenta arrows in Fig. 5b and the enlarged image Fig. 5e. After stored for 4 h (Fig. 5c), the ordered arrangement of myelin sheaths is severely disrupted. The splitting of individual myelin lamellae becomes common and the majority of the disrupted sheaths appear as loosened myelin membranes surrounded by myelin debris, see Fig. 5c and f, indicating severe degeneration in the tissue. Such demyelination may be due to loss of mature oligodendrocytes and/or loss of trophic support after axonal degeneration^42^.

Most cells in the human body contain mitochondria, the main function of which is to metabolize or break down carbohydrates and fatty acids in order to generate energy. As such mitochondria are commonly known as the powerhouses of the cell. These powerhouses produce adenosine triphosphate (ATP), a molecule which transports chemical energy within the cell to fuel other cellular processes. This, along with other functions including the synthesis of proteins, indicates that mitochondria are essential for normal brain functions^43^.

Thus, it is not surprising that an impairment of mitochondrial function results in cellular damage and is linked to neurodegeneration. Many existing studies suggest that mitochondrial dysfunction plays a central role in several neurodegenerative diseases, including Parkinson’s disease and Alzheimer’s disease^44^. Siesjö^45^ reported that the high-energy phosphate levels had virtually disappeared (ATP depletion) within 5 minutes’ interruption of blood flow to the mammalian brain. For this reason, we analyzed the degeneration in the morphology of mitochondria in the axons and astrocytes with the prolonged storage duration. The TEM micrographs of mitochondria in axon and astrocyte are shown in Fig. 5g–m. Figure 5g and j demonstrate that, after storing the tissue for 0 h, mitochondria are bounded by a double-membrane system, consisting of the outer membrane (indicated by the blue arrow) and the folded inner membrane (also known as cristae, indicated by the magenta arrow). After stored for 1 h (Fig. 5h and k), no obvious degeneration is observed and both of these two membranes are still visible. However, after 4 h storage (Fig. 5i and m), the mitochondria exhibit abnormal morphology. The dilatation and vacuolization of cristae are obvious and the cristae cannot be clearly identified, indicating the degeneration of mitochondria. This degeneration may be attributed to the profound disturbances in cell electrolyte balance: potassium begins to leak rapidly from the intracellular compartment and sodium and calcium begin to enter the cells^46^. Sodium influx results in a significant increase in the cellular water content^47^.

The results in this study reveal that after 4 h storage, the myelin lamellae and mitochondria are severely damaged, which suggests that the brain tissue is highly vulnerable to ischemia and hypoxia. This is consistent with the measured stiffness shown in Figs 2 and 3: the engineering stress decreases with prolonged storage duration. These findings also agree well with the literature. Weichkenmeier *et al*. reported that the brain tissue stiffness is correlated to the underlying tissue microstructure and directly proportional to the local myelin content. The cerebral white matter stiffness increases with increasing myelin content^48,49^. Shreiber *et al*. found that the demyelinated spinal cords demonstrate significantly lower stiffness and ultimate tensile stress than myelinated spinal cords^50^.

As stated in the introduction section, various conditions have been used to store brain tissue for *in vitro* measurement of the stiffness of brain tissue and there are no standard guidelines available for the storage conditions^51,52^. In existing studies, brain tissue has been stored either in liquid media such as cerebral spinal fluid^53^, artificial cerebrospinal fluid^24,54–56^, phosphate buffered saline^19–23^ and physiological solution^25–27,57,58^ or without any liquid^11,18,59^. The storage duration varies from minutes to days and the temperature varies from ice-cold to body temperature. The TEM analyses here demonstrate that myelin sheaths degenerate only slightly and no pronounced degeneration in mitochondria appear after 1 h storage of brain tissue, which indicates that, if the brain is stored at 1 °C without any liquid, it is better to control the storage duration to be less than 1 hour.

It needs to be pointed out that the stiffness and thermal property differences of brain tissue from 1 h to 4 h are not significant, while TEM shows significant variation for 4 h. Further work is necessary to quantitatively correlate the change of stiffness and thermal property with the variation of organelles.

## Conclusion

For brain tissue stored at 1 °C without any liquid medium, the mechanical strength of both white and grey matter deteriorates with prolonged storage duration. The DSC analysis on brain tissue thermal behavior indicates a decrease in both the conformation transition temperature and the entropy of white and grey matter as the storage duration is increased. Further DSC analysis eliminates the possible damaging effect due to the formation of ice. TEM observations suggest the degeneration of myelin sheaths and the vacuolization of cristae with prolonged storage duration.

The prolonged storage duration for brain tissue would cause damages to the cross-linking density of bio-molecule, the micro-structure of myelin sheath and mitochondria, and the structure of nucleus. Although currently it is still difficult to quantitatively correlate the damages at the micro level to the variations of mechanical properties at the macro-scale level, this study lays the groundwork for analyzing the effect of *in vitro* storage conditions on the mechanical response of brain tissue. This study can be further extended to include the storage medium for the purpose of comparing the effect of commonly used media (such as cerebral spinal fluid, artificial cerebrospinal fluid, phosphate buffered saline and physiological solution) on the mechanical properties of brain tissue. The results from this study might help resolve the discrepancies in literature and lead to a more accurate characterization of the mechanical properties of brain tissue.

## Materials and Methods

### Sample preparation

Small-tailed Han sheep (kindly provided by Dalian Shun Feng Farm, China) were used. These sheep were at the same age (1.5 y) and were raised in the same manner. After euthanasia, the sheep were dissected and their mechanical property tests were completed within 30 mins, representing the storage duration of 0 h. For the mechanical property measurements after 1 h and 4 h storage, the brain tissue samples were stored in a refrigerator at the temperature 1.0 ± 1.0 °C for 1 hour and 4 hours, respectively. All experiments were carried out in accordance with the relevant guidelines and regulations of the Biological and Medical Ethics Committee of Dalian University of Technology, China, and all experimental protocols were approved by this committee.

### Mechanical property measurement

**Figure 6:**
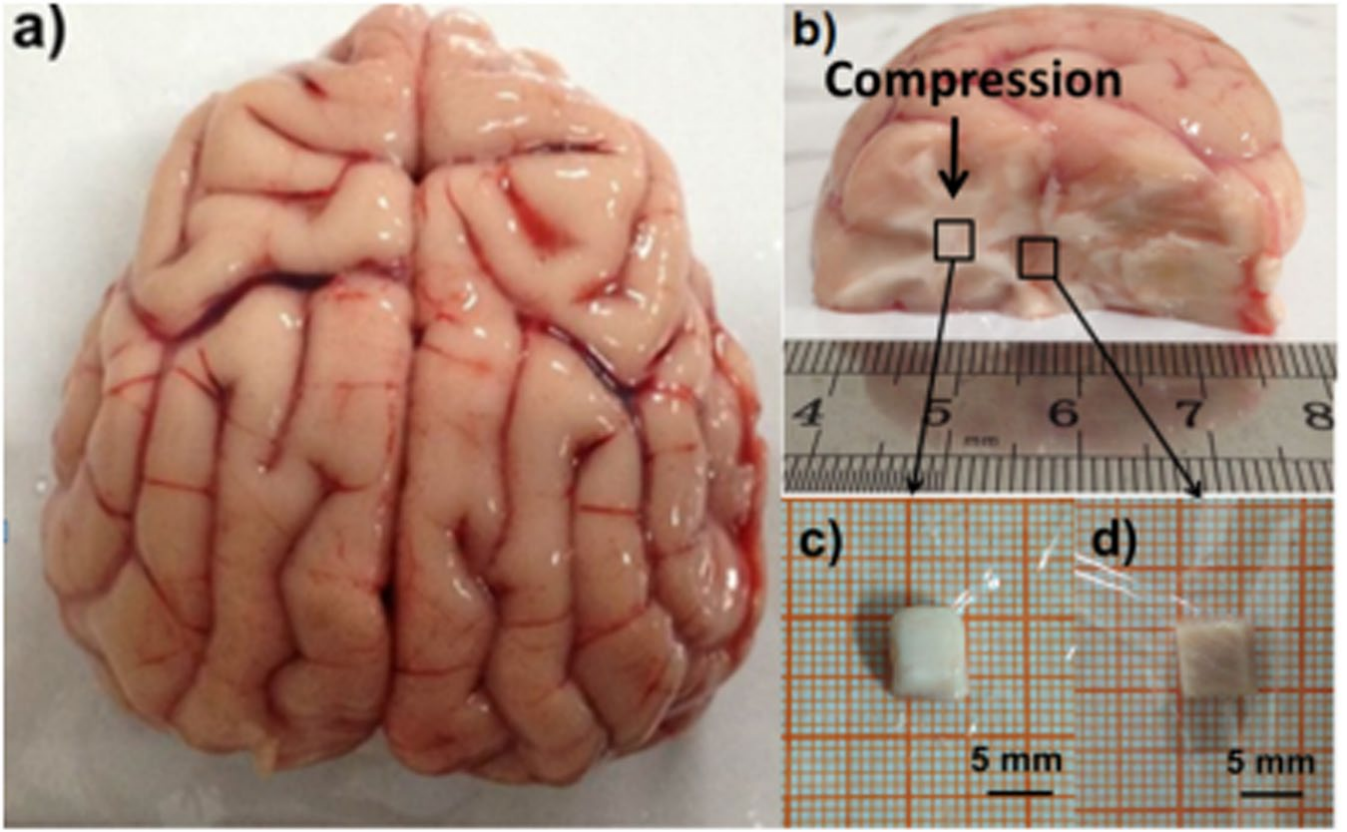
An example of the sample used in this study. (**a**) The whole brain; (**b**) a half brain cut along the vertical plane; (**c**) white and grey tissue samples for mechanical property tests.

Using methods developed by the authors previously^60,61^, pure white and grey matter samples with the size of 5 mm × 5 mm × 5 mm (±0.1 mm) were taken from the corona radiate and thalamus, respectively, as illustrated in Fig. 6. To control the storage time as accurate as possible, only one sample, either white or grey, was taken from each brain. In total, 30 samples (15 white and 15 grey) were obtained from 30 brains. For each level of the storage duration, 5 samples were tested to represent the repeatability of the analysis.

The uniaxial compression experiments were performed on the white matter along the direction shown in Fig. 6. For the grey matter, the sample was compressed with no preference about the direction. The top plate was mounted to a 50 N capacity load cell with sensitivity of 1 mN (BAB S type aluminium weighing sensor, BAB-XS-5M, Transcell Technology Inc., USA), which was attached to the actuator of a 20 kN Sans Universal Testing Machine (Shenzhen SANS Testing Machine Co., Ltd., CMT-4204, China). The sampling frequency for both the force and displacement data was 100 Hz. Prior to testing, the top and bottom plates were lubricated with physiological saline (0.9% NaCl) to approximate a nearly pure slip condition. In compression test, the top plate was slowly lowered until it almost touches the sample. The starting point of the compression test was defined as the time that the force reached 1 mN. Each sample was tested once and then discarded. All tests were performed at room temperature (21 ± 1.5 °C). During experiments, physiological saline was regularly sprayed onto the specimen to prevent dehydration and to slow down degradation of the tissue.

### DSC measurement

DSC measurements were conducted on a Netzsch DSC-204 calorimeter (Germany). The samples of white and grey matter (weighing 5–10 mg) were taken from the corona radiate and thalamus of the brain, respectively (see Fig. 6). For each brain, either one white matter sample or one grey matter sample was obtained. In total, 8 samples (4 white matter samples and 4 grey matter samples) were taken from 8 brains. The obtained white/grey matter samples were sealed in standard aluminium pans (volume 40 μl) and an empty pan was used as reference. In characterizing the effect of storage duration on the thermal behavior of brain tissue, the samples were cooled to 1 °C from room temperature at 5 °C/min and the temperature was maintained at 1 °C for 0 h, 1 h and 4 h. After the storage experiment, the temperature was increased to 60 °C at 5 °C/min. In the characterization of ice formation, the samples were cooled to −100 °C from room temperature at 5 °C/min and then the temperature was increased to 10 °C at 5 °C/min. Liquid nitrogen was used to cool the specimens to lower than room temperatures.

### TEM observation

TEM slides were prepared in reference to Li’s method^62^. In brief, glutaraldehyde-fixed brain tissues were fixed in osmic acid, dehydrated in ethanol, and then embedded. The tissue was sectioned into 0.05μm slices and stained with uranyl acetate and lead citrate. These slices were then examined on a transmission electron microscope (JEOL JEM-2000EX, Japan). Four white matter samples and four grey matter samples were examined and the representative images are shown in Fig. 5.

### ANOVA and pairwise comparison

One-way and two-way ANOVA were performed in SPSS. The Fisher’s Least Significant Difference (LSD) test was utilized for the pairwise comparisons and also performed in SPSS.
